# Study on the strength deterioration characteristic and damage model of coal pillar dams with repeated water immersion in underground reservoirs

**DOI:** 10.1038/s41598-024-56741-8

**Published:** 2024-03-15

**Authors:** Beifang Wang, Duo Zhou, Jing Zhang, Bing Liang

**Affiliations:** 1 School of Mines, Liaoning Technical University, Fuxin, 123000 Liaoning China; 2 State Key Laboratory of Water Resource Protection and Utilization in Coal Mining, Beijing, 102299 China; 3 Shandong Key Laboratory of Mining Disaster Prevention and Control, Shandong University of Science and Technology, Qingdao, 266590 China; 4 School of Science, Liaoning Technical University, Fuxin, 123000 Liaoning China; 5 School of Mechanics and Engineering, Liaoning Technical University, Fuxin, 123000 Liaoning China

**Keywords:** Water immersion times, Coal pillar dam, Mechanical properties, Deterioration, Acoustic emission, Damage, Engineering, Environmental impact

## Abstract

The continuous operation of coal mine underground reservoirs exposes the coal pillar dams to mining disturbances and prolonged water immersion, resulting in the deterioration of coal pillars' mechanical properties and posing a serious threat to the dam stability. To this end, coal samples from the proposed pillar dam in the 5–2 coal seam of Daliuta Mine in Shendong Mining Area were selected for conducting water absorption tests and triaxial compression tests under conditions of repeated water immersion, in order to study the deterioration of the mechanical properties and acoustic emission damage characteristic of coal samples as well as the mechanism behind the deterioration of coal samples under the water–rock interaction. The results indicated that: (1) the saturated water content of coal samples exhibited a progressive increase as the water immersion times increased, but with a diminishing rate of growth. (2) As the water immersion times increased, the compressive strength, cohesive force, and internal friction angle of coal samples gradually decreased. Notably, the deterioration effect was more pronounced in compressive strength and cohesive force, while the decline in internal friction angle was relatively minor, and the total deterioration degree and the stage deterioration degree of the above three had evident cumulativity and non-uniformity. The progressive rise in water immersion times led to a gradual attenuation of the deterioration effect. Meanwhile, the confining pressure exhibited a certain inhibitory impact on the strength deterioration of coal samples. (3) Compared to the dry coal samples, the average AE count rate of coal samples subjected to a single water immersion exhibited a significant decrease, and subsequent water immersion for two, three, and four times resulted in a very minor decrease in the average AE count rate. (4) The AE cumulative ringing counts in coal samples exhibited varying degrees of reduction as water immersion times increased. Specifically, the most significant decrease in AE cumulative ringing counts occurred after the initial water immersion, followed by a gradual decrease thereafter. The energy-releasing capacity of coal samples decreased, while their plasticity exhibited a gradual increase. (5) A damage model was developed for coal samples based on the water immersion times. The model indicated that the damage to coal samples increased as the water immersion times increased, and the damage rate gradually decreased and eventually stabilized. (6) The deterioration mechanism of coals under the water–rock interaction was explained. Through repeated water immersion, the physical, chemical, and mechanical interactions between water and coal induced alterations in the internal microstructure of coal samples, resulting in the deterioration of mechanical properties such as compressive strength, cohesive force, and internal friction angle, which was a cumulative damage process from the microscopic to the macroscopic level.

## Introduction

**Figure 1:**
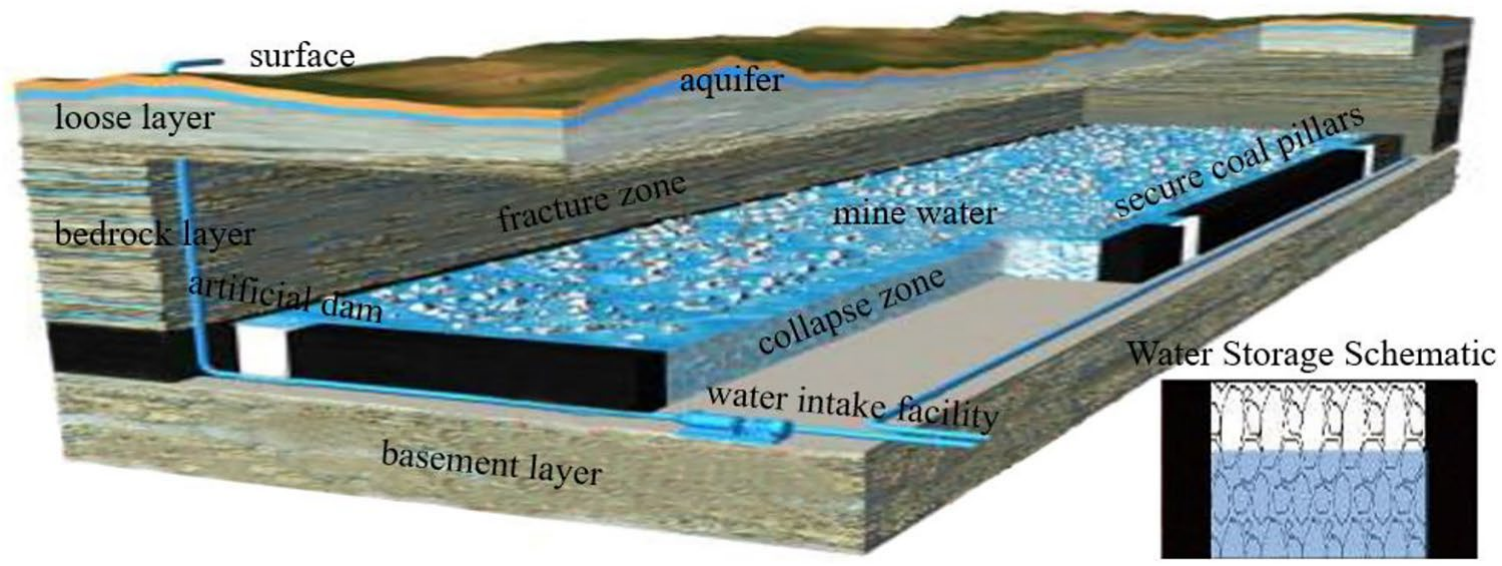
Schematic diagram of the coal mine underground reservoir.

With the full implementation of coal mining westward strategy, Shendong mining area has gradually developed into China's mega-coalfield development base, but it is situated in an arid-semi-arid zone and has a shallow coal seam. The large-scale and high-intensity coal mining has left a large area of goaf, mining-induced cracks run through the surface, and aquifers suffer massive water loss, which further aggravates the problem of water shortage in the mining area^1–5^. Hence, academician Gu Dazhao et al.^6^ innovatively proposed the use of the goaf to establish underground reservoirs for water storage technology. This technology realizes the recovery, storage and utilization of mine water by utilizing the voids formed by collapsed coal and rock blocks in the goaf as water storage spaces, constructing artificial dams connected secure coal pillars to serve as the water retaining dams, and allocating water injection and intake facilities, as shown in Fig. 1. The coal mine underground reservoir project has effectively alleviated the serious shortage of water resources in arid and semi-arid mining areas in western China. However, during the long-term operation of underground reservoirs, the water within the storage region repeatedly corrode the coal pillar with the fluctuation in water levels, so that the coal pillar dam experiences a cyclic process of water immersion, water loss, and water immersion again, leading to a gradual deterioration in the mechanical properties and stability of the coal pillar. Therefore, it is imperative to conduct research on the impact of water immersion times on the deterioration characteristics of mechanical properties in coal, which is of great significance to the water storage structure construction and stability evaluation in coal mine underground reservoirs.

A lot of research has been dedicated to the physical properties deterioration and damage characteristic of coals and rocks under the water–rock interaction by domestic and foreign scholars. Poulsen et al.^7^ quantified the strength reduction of coals between unsaturated water content and saturated water content by physical tests. Alexeev et al.^8^ investigated the variation of strength of water-saturated coal with water content under true triaxial stress conditions. Perera et al.^9^ compared the strength and deformation characteristics of water-saturated and dry coal samples. Vishal et al.^10^ researched the acoustic emission characteristics of fluid coal samples with different saturations, and obtained the relationship between water content and the uniaxial compressive strength of coal. Yao et al.^11^ explored the variation in mechanical parameters such as uniaxial compressive strength and elastic modulus of coal samples under different water content conditions, and analyzed the damage evolution characteristics of water-bearing coal samples after loading by combining with acoustic emission counting. Liu et al.^12^ conducted the water injection test on the coal and analyzed the effect of water content on the uniaxial compressive strength and elastic modulus after water injection. Chen et al.^13^ carried out acoustic emission tests under uniaxial compression on five groups of coal samples, and examined the development and damage characteristics of fracture about coals when subjected to repeated water immersion. Zhu et al.^14^ implemented water content effect tests on the strength characteristic of soft coal at varying levels of porosity, and obtained the variation of these coals' strength under the combined effect of water content and porosity. Wang et al.^15^ analyzed the stress–strain characteristics and damage mechanisms of raw coal and briquette coal with different water content during uniaxial compression, and established a segmented statistical constitutive model about coal damage based on water content. Mao et al.^16^ analyzed the correlation between burst tendency of coal or rock, and water content by conducting loading tests on coal samples with different water content. Yang et al.^17^ used the Split Hopkinson Pressure Bar (SHPB) test equipment to perform dynamic splitting experiments, and obtained the characteristic that the energy dissipation property of coal samples change with water content. Yang et al.^18^ studied the creep properties of strip coal pillars with different water content by using Flac3D numerical simulation software based on the creep test results of coal samples with different moisture content. Wei et al.^19^ observed the microstructure and pore characteristic of the remolded loess and undisturbed loess using electron microscope observation and nuclear magnetic resonance testing, and concluded that the mesoscopic mechanism of loess thixotropic strength recovery was that the connection between soil particles was re-established after the break of the clay particle-water-charge system. Chen et al.^20^ carried out uniaxial loading tests on coal-rock combined samples with five kinds of water immersion time, and investigated the mechanical properties, energy and damage characteristics of coal-rock combined samples under water–rock interaction. Xie et al.^21^ studied the mechanical properties and microstructure of sandstone at different pH, Na_2_SO_4_ solution concentration and soaking time, and found that hydrochemical action has a significant effect on the erosion or degradation of the cemented sandstone. Liu et al.^22^ found through experimental studies that the deterioration effects of different water–rock interactions on rocks were generally characterized by thermal wet cycle > dry wet cycle > long-term immersion. Wang et al.^23,24^ studied the fracture characteristics, acoustic emission features and energy evolution patterns of granite under different cooling cycling conditions. Luo et al.^25^ established a PFC sandstone creep model to study the crack initiation, extension, penetration and energy evolution of sandstone under water–rock interaction.

For the safety of coal mine underground reservoirs, many scholars have conducted research around the stability, structure and size of the dam. Bai et al.^26^ researched on the relationship between the stability of the reservoir and the critical hydraulic pressure of the coal pillar, and theoretically calculated the limit head value of underground reservoir. Wang et al.^27^ used FLAC 3D to construct a dynamic damage numerical model of coal pillar dam in multi-face mining, and analyzed the failure evolution characteristic of coal pillar dams in complex stress environment. Chi et al.^28^ investigated the variation and failure precursor information of coal pillar dams with different sizes under vertical and horizontal pressure. Wu et al.^29^ analyzed the influence of mine vibration load at different focal locations on the coal pillar dam and artificial dam of the underground coal mine reservoir by using numerical simulation. Wang et al.^30^ analyzed the dynamic evolution and mechanism of water inrush in karst roofs under different mining sequences by constructing a similar model, proposed an upward-inclined mining method to control water inrush in the roof, and found that the water in the goaf of the lower coal group was more suitable for water resource utilization. Li et al.^31^ studied influence of coal mine underground reservoir water storage soaking to coal pillar dam strength through immersion experiments. Zhou et al.^32^ studied the secondary expansion rule of the cracks in the plastic zone of the coal pillar dam under the action of water storage pressure through numerical simulation, and determined the minimum retention width of the coal pillar dam.

The above experts found a clear deterioration trend of coal and rock under water–rock interaction, and analyzed the dam stability of underground reservoir under different influencing factors. These studies generally focused on the deterioration effect of long-term water immersion on coal and rock mechanics. However, during the use of the underground reservoir, the drainage and storage will inevitably lead to the change of water level, and the coal pillar dam is periodically in the alternating state of unwatering and saturation. It is closer to the real environmental changes of underground reservoirs to carry out relevant research with coal samples of pillar dams under repeated water immersion, which has rarely been reported. Accordingly, the author takes the typical coal samples of Daliuta Coal Mine as the research object and conducts triaxial compression tests on coal samples with different water immersion times, supplemented by acoustic emission technology monitoring, to research the deterioration rule of physical properties and acoustic emission damage characteristics of coal under repeated water immersion and elucidate the mechanism by which coals deteriorate under water–rock interaction. The research results can provide theoretical and technical support for the construction and safety and stability analysis of coal pillar dams in underground reservoirs.

## Engineering overview

Daliuta Coal Mine is a mega backbone mine in Shendong mining area, located at the edge of the Maowusu Desert, with a typical semi-arid, semi-desert plateau continental climate and extremely scarce water resources. For this reason, Daliuta Coal Mine daringly constructed an underground reservoir for the first time, and successfully realized the purification and recycling of mine water^33^. Daliuta Coal Mine's 2–2 coal seam at the first mining level and 5–2 coal seam at the second mining level have a spacing of 155 m. At present, the 2–2 coal seam has been mined out, and three underground reservoirs have been built by making full use of space in the goaf. No. 4 underground reservoir under construction is located in the goaf of 52,302–52,307 working face in the third panel of 5–2 coal seam. Taking 52,307 fully-mechanized face as an example, it is 301 m wide by 4462.6 m long with the dip angle of 1°–3°. The coal seam is 7.1–7.4 m thick with an average of 7.2 m and has 1–2 layer partings with a thickness of 0.2 m. The coal seam structure is simple, and the mining operation employs single-incline longwall backward comprehensive mechanized mining and treats the goaf through the total caving method. The working face corresponds to the surface elevation of 1120.2–1217.1 m, mostly covered by quaternary aeolian sand. The bedrock layer is 135–165 m thick, and the loose layer that covers it is 0–20 m thick. Siltstone and fine-grained sandstone make up the majority of the bedrock layer. During the operation of the underground reservoir, mine water will cause repeated immersion damage to the boundary of the underground reservoir. The strength of coal pillar dam is weakened seriously and the safety is reduced. Therefore, it is necessary to investigate the strength variation and damage evolution characteristics of the coal pillar dam under repeated water immersion, in anticipation of providing reference for the width retention and safety evaluation of coal pillar dams in underground reservoirs after construction.

## Method

### Test preparation

1. Sample preparation.
  The samples were selected from the 5–2 coal seam of Daliuta Coal Mine of Shendong Coal Group, Shaanxi Province, as shown in Fig. 2. According to GB/T 2356.1-2009 "Methods for Determination of Physical and Mechanical Properties of Coal and Rock, Part 1: General Provisions for Sampling", coal blocks with favorable integrity and homogeneity were cored and transformed into square samples measuring 50 mm × 50 mm × 50 mm, among which samples with relatively uniform dense structure and devoid of any surface cracks or deformations were chosen. The selected samples underwent a sandpaper treatment to ensure smoothness on their end faces. Among them, the end face of coal samples should be perpendicular to the axis, the maximum deviation should be less than 0.25°, and the error of non-parallelism of the two end faces should be less than 0.05 mm.

**Figure 2:**
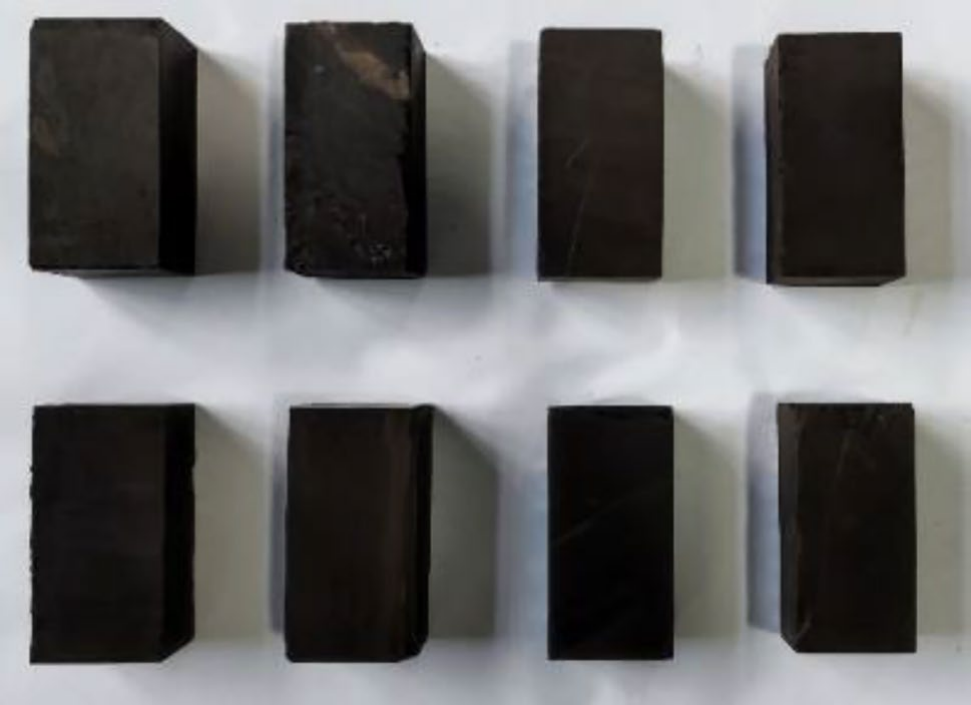
Schematic diagram of some standard coal samples.
2. Water absorption test for coal samples.
  Considering the anisotropic characteristic of coal samples and the influence of water level fluctuation in underground reservoirs on the water absorption characteristic of coal pillar dams, with reference to the preparation method of saturated coal samples described in Xiong et al.^34^, mine water was collected on site to carry out repeated immersion tests of coal bodies. Mine water is a complex aqueous solution containing complex ionic compositions, usually acidic or alkaline, and the corrosion degree of coal and rock by mine water of different acidity or alkalinity is in the following order: acidic > alkaline > neutral^35^. Due to the large differences in the water richness as well as the mineralization of mine water in different areas, in order to determine the degree of hydrochemical action on the coal pillar dam, the mine water composition in Daliuta Coal Mine was tested and analyzed in accordance with the "water quality sampling program design technical provisions (HJ 495-2009)" and "groundwater quality test methods (DZ/T 0064-1993)". The results show that Cl HCO-K Na type water quality characterizes the mine water, which is neutral to slightly alkaline, so dams are less exposed to chemical corrosion. The details are shown in Table 1. The composition of mine water is closely interconnected with water–rock interaction processes such as adsorption, erosion, and redox reactions. The primary source of HCO^3−^ and Na^+^, respectively, is the dissolution of carbonate minerals and rock salts.

**Table 1:** Mine water composition.

Test indicators	K^+^, Na^+^ (mg L^−1^)	Ca^2+^ (mg L^−1^)	Mg^2+^ (mg L^−1^)	Cl^−^ (mg L^−1^)	SO_4_^2−^ (mg L^−1^)	HCO^3−^ (mg L^−1^)	pH (dimensionless)
Test results	20.01–425.96	34.47–55.31	2.43–23.81	4.0–555.0	14.0–100.0	165.97–268.47	7.40–11.66
  To simulate the progressive effect of weathering and water immersion on the deterioration of coal pillar dams under natural conditions, coal samples were subjected to five distinct working conditions: "dry", "soaked once", "soaked twice", "soaked three times", and "soaked four times". Firstly, the first group of coal samples would be put into the vacuum drying oven to dry for 24 h, as shown in Fig. 3a. The drying temperature was 105 ℃. This process was used to get "dry" coal samples. Next, the second group of coal samples was subjected to a water saturation treatment by drying them and placing them in a water tank. Mine water was added every 2 h and coal samples were submerged after 5 additions, as shown in Fig. 3b. The samples were saturated after 120 h of immersion by default, after which they were removed and gently wiped to eliminate any water droplets on the surface, resulting in the acquisition of the "soaked once" coal samples. Similarly, coal samples of "soaked twice", "soaked three times", and "soaked four times" could be created, and the specific implementation steps were shown in Table 2. During the experimental procedure, the coal samples were weighed both before and after each soaking period, and the saturated water content of coal samples with different immersion times was determined by Eq. (1).
  1 $$W = \frac{{G - G_{0} }}{{G_{0} }} \times 100\%$$
  where: *W* is the saturated water content of coal samples, %; *G* is the mass of coal samples after saturated water treatment, g; *G*_0_ is the initial mass of dry coal samples, g.

**Figure 3:**
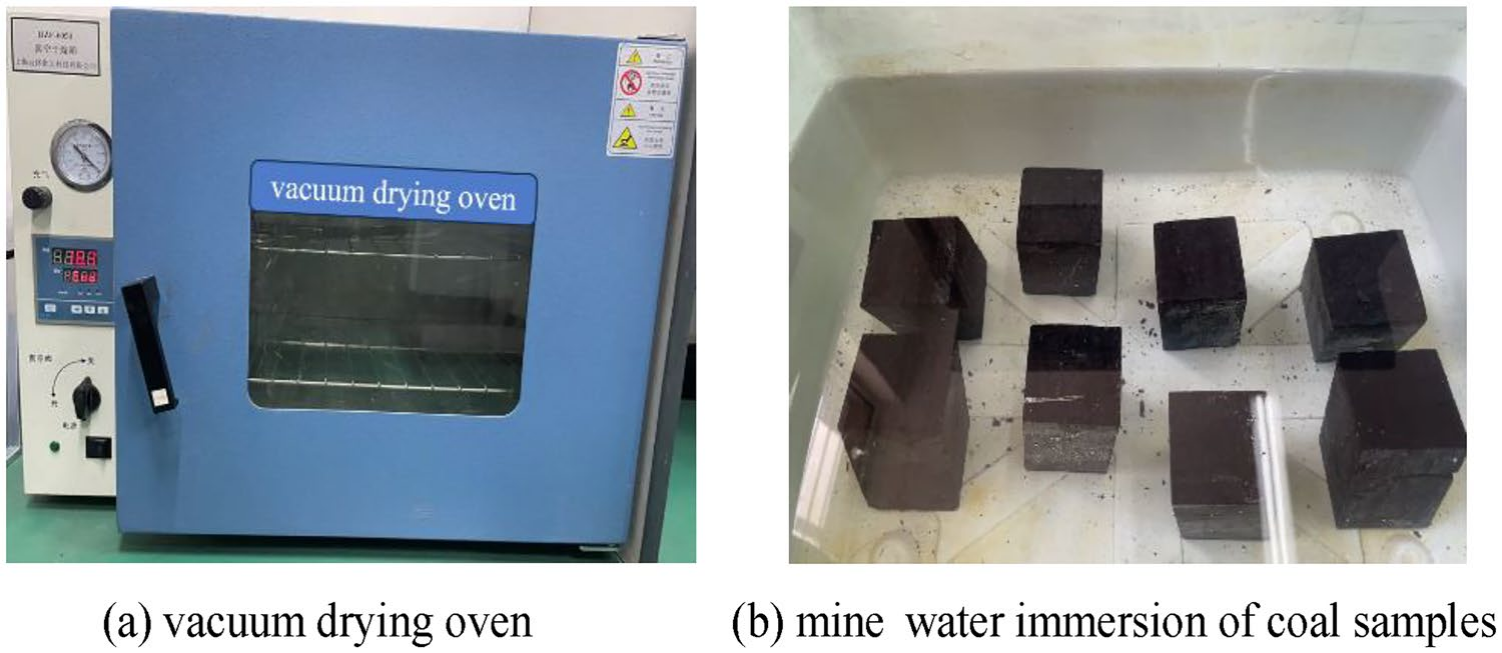
Schematic diagram of water absorption test.

**Table 2:** Repeated water immersion steps for coal samples.

Group	Step 1	Step 2	Step 3	Step 4	Step 5	Step 6	Step 7	Step 8
Dry	Dry	–	–	–	–	–	–	–
Soaked once	Dry	Soak to saturation	–	–	–	–	–	–
Soaked twice	Dry	Soak to saturation	Dry	Soak to saturation	–	–	–	–
Soaked three times	Dry	Soak to saturation	Dry	Soak to saturation	Dry	Soak to saturation	–	–
Soaked four times	Dry	Soak to saturation	Dry	Soak to saturation	Dry	Soak to saturation	Dry	Soak to saturation
  The water absorption of coal is one of its important hydrological properties, determined by factors such as the quantity and dimensions of its open pores, the arrangement of mineral particles within the coal, its susceptibility to moisture, and the air elimination from the pores. The smaller the water absorption is, the harder and denser the coal surface is, and the better its engineering properties. Figure 4 illustrates the variation in water absorption of coal samples as a function of the water immersion times. The water content after being immersed in water for 1, 2, 3, and 4 times was recorded as 9.04%, 9.67%, 9.90%, and 10.03%, respectively. The saturated water content of coal grew logarithmically with the increase of water immersion times when subjected to repeated water immersion. The rate of increase in water content of coal was more pronounced during the initial phases of the water immersion process, gradually diminishing in subsequent stages. The increment of the stage slowed down as the water immersion times increased. It can be attributed to the initial water immersion of coal samples, which led to the development of primary fissures within the samples, so the role of initial water absorption on the deterioration of coal mechanical properties became more pronounced, and the later damage gradually weakened and eventually reached a stable state.

**Figure 4:**
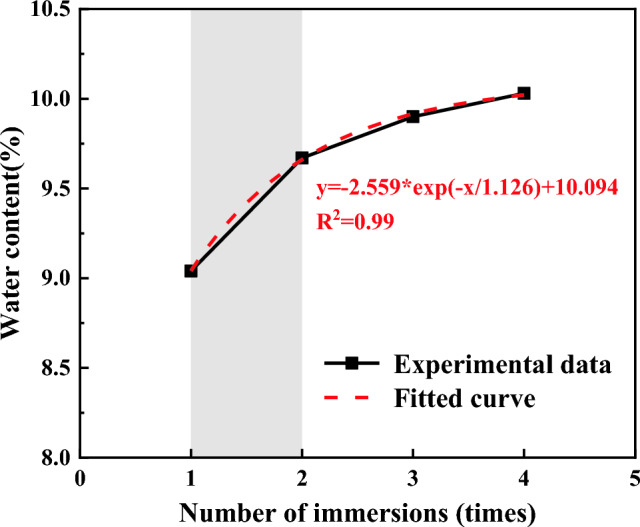
Curve of saturated water content of coal samples versus the number of water immersions.

### Test loading programme

**Figure 5:**
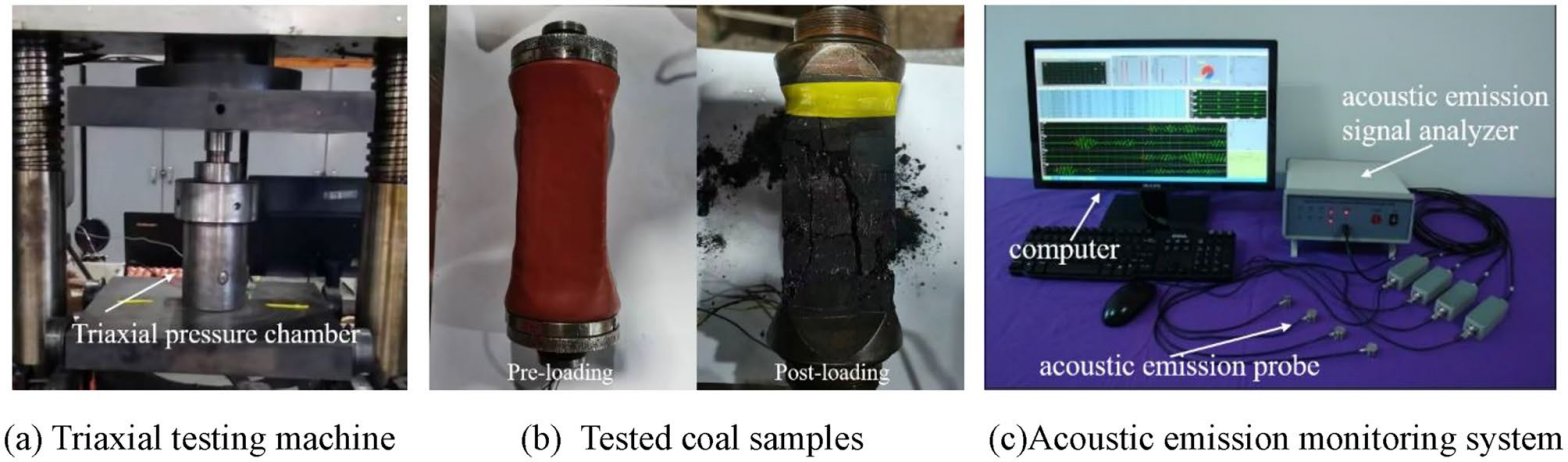
Test system.

This test was mainly a triaxial compression test using coal with different water immersion times. The test system was shown in Fig. 5. The TAW-2000 microcomputer-controlled electro-hydraulic servo rock triaxial testing machine was used to conduct the test. The experimental system can withstand a maximum axial load of 1000 kN and a maximum confining pressure of 60 Mpa. Its displacement sensor can measure displacement with a testing accuracy of 0.5% Fs. The test equipment consisted of three loading systems: axial, lateral, and microcomputer operating. Each loading surface of the coal and rock triaxial can have its pressure and displacement individually controlled.

Acoustic emission refers to the phenomenon of transient elastic wave generated by the localized rapid release of energy when the material is subjected to external or internal forces. Coal, as a typical brittle material, is loaded along with fissure expansion, forming damage or even failure. While in the damage evolution stage of coal, the acoustic emission instrument can effectively monitor the elastic stress wave release process, and reflect the damage evolution condition of coal interior by processing, analyzing and researching the acoustic emission signals. Acoustic emission monitoring system in this test adopts the DS5-16B full-information acoustic emission signal analyzer. System components include the main control computer, transducer, preamplifier, supply signal separator, external filter, attenuator, A/D converter, signal line and signal generator, which can realize the functions including signal acquisition, parameter extraction, waveform playback, data storage, graphic display, etc., and it can be used for the simultaneous acquisition of 16-channel acoustic emission signals and waveform acquisition.

Six groups of coal samples were processed. One group was kept as a reserve, while the remaining five groups of coal samples were dried and subjected to 1, 2, 3, and 4 times of water absorption treatment, respectively. The samples were mounted and loaded at a rate of 0.025 MPa/s to the predetermined values, with the confining pressure values set at 2, 4, 6, and 8 MPa. Following the test, displacement control was used to load and maintain a consistent confining pressure. After the confining pressure loading was complete, maintain the confining pressure while increasing the axial pressure by displacement control loading until the samples ruptured and destabilized, at which point the displacement loading rate of axial pressure should be 0.02 mm/s. Acoustic emission signals accompanying the coal break-up process need to be captured immediately. The sampling frequency was set at 1 MHz with a threshold value of 40 db.

## Results

### Deterioration law of physical properties of coal after repeated water immersion

**Figure 6:**
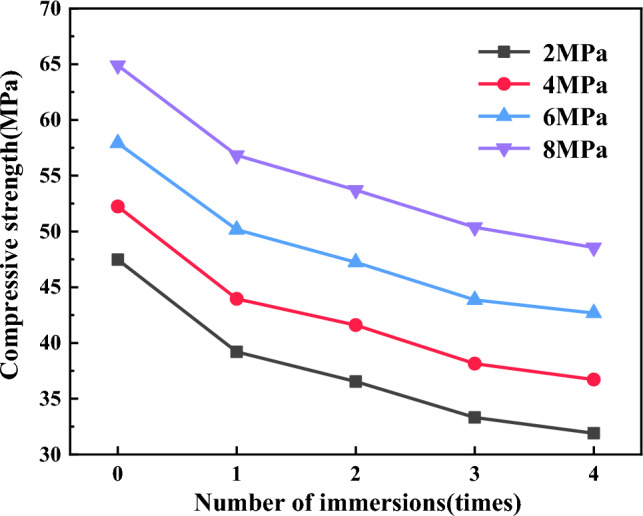
Graph of compressive strength of coal samples under different water immersion times.

**Figure 7:**
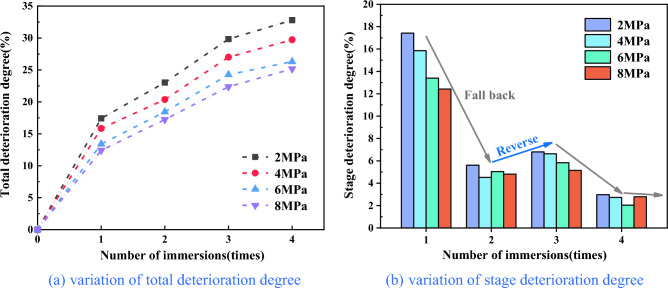
Diagram of deterioration degree of the compressive strength of coal samples.

1. Analysis of the deterioration law of the compressive strength of repeatedly immersed coal.
  The deterioration degree is defined as the extent of reduction of mechanical parameters in coal samples during repeated water immersion. It serves as an indicator of the damage degree to coal and rock under water–rock interaction. The total deterioration degree represents the overall reduction in mechanical parameters of coal samples before and after water immersion, which can be expressed as:
  2 $$S_{i} = \frac{{T_{0} - T_{i} }}{{T_{0} }} \times 100\%$$
  where: *S*_*i*_ is the total deterioration degree of coal samples after repeated water immersion, %; *T*_0_ is the initial mechanical parameters of coal samples before water immersion, including compressive strength, cohesive force and internal friction angle, etc.; *T*_*i*_ is the mechanical parameters of coal samples at different water immersion times during repeated water immersion process.
  To better characterize the effect of repeated water immersion processes on the mechanical properties of coal samples, focusing on the initial mechanical parameter *T*_0_ as the baseline, the deterioration of a single water immersion action is defined as the stage deterioration degree Δ*S*_*i*_, which can be expressed as:
  3 $$\Delta S_{i} = S_{i} - S_{i - 1}$$
  The test results of compressive strength of coal samples under different water immersion times were shown in Table 3. To analyze more visually the deterioration characteristics of the compressive strength of coal samples during repeated water immersion, Figs. 6, 7 and 8 was made.

**Table 3:** Compressive strength and deterioration degree of coal samples with different water immersion times.

Time	Confining pressure											
	2 MPa			4 MPa			6 MPa			8 MPa		
	R_c_ (MPa)	S_i_ (%)	ΔS_i_ (%)	R_c_ (MPa)	S_i_ (%)	ΔS_i_ (%)	R_c_ (MPa)	S_i_ (%)	ΔS_i_ (%)	R_c_ (MPa)	S_i_ (%)	ΔS_i_ (%)
0	47.47	0	0	52.24	0	0	57.92	0	0	64.89	0	0
1	39.20	17.42	17.42	43.96	15.85	15.85	50.16	13.40	13.40	56.83	12.42	12.42
2	36.54	23.03	5.61	41.60	20.37	4.52	47.24	18.44	5.04	53.71	17.23	4.81
3	33.31	29.83	6.80	38.14	27.00	6.63	43.86	24.27	5.83	50.37	22.38	5.15
4	31.90	32.80	2.97	36.71	29.73	2.73	42.68	26.31	2.04	48.56	25.17	2.79

**Figure 8:**
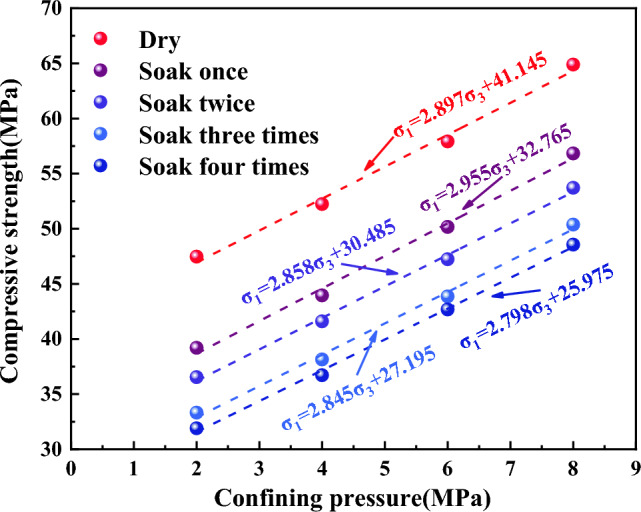
Relationship between confining pressure and compressive strength of coal samples.
  Figure 6 illustrates the relationship between the compressive strength of coal samples and the number of water immersions, considering four different confining pressure conditions. From Table 1 and Fig. 6, it can be seen that the compressive strength of coal samples immersed in water from 0 to 4 times with a confining pressure of 2 MPa were 47.47 MPa, 39.20 MPa, 36.54 MPa, 33.31 Mpa and 31.90 MPa, respectively, in which water immersion for 1 time and 4 times decreased by 17.42% and 32.80% respectively compared with that before water immersion; the compressive strength of coal samples with a confining pressure of 4 MPa were 52.24 MPa, 43.96 MPa, 41.60 MPa, 38.14 MPa and 36.71 MPa, respectively, in which water immersion for 1 time and 4 times decreased by 15.85% and 29.73% respectively compared with that before water immersion; the compressive strength of coal samples with a confining pressure of 6 MPa were 57.92 MPa, 50.16 MPa, 47.24 MPa, 43.86 MPa, and 42.68 MPa, respectively, in which water immersion for 1 time and 4 times decreased by 13.40% and 26.31% respectively compared with that before water immersion; the compressive strength of coal samples with a confining pressure of 8 MPa were 64.89 MPa, 56.83 MPa, 53.71 MPa, 50.37 MPa, and 48.56 MPa, respectively, in which water immersion for 1 time and 4 times decreased by 12.42% and 25.17% respectively compared with that before water immersion. It has been shown that the compressive strength of coal samples decreased with an increasing number of water immersions under various confining pressure. This consistent trend indicated that the compressive strength of coal samples was negatively affected by repeated water immersions. The compressive strength of coal samples exhibited varying degrees of decline as the water immersion times increased. The most significant reduction in compressive strength occurred after a single water immersion, after which the rate of decrease gradually slowed down.
  The reason is that coal itself is a non-homogeneous and multi-defective material composed of multiple minerals of different sizes and shapes cemented together. The water and hydrophilic material present in coal were prone to physicochemical interaction, leading to phenomena such as displacement, dissolution, ion exchange and secondary minerals, and the internal microstructure of the coal was changed. When a water-saturated coal sample was subjected to compression, it induced pore water pressure within certain imperfections. This pore water pressure exerted additional stress on the main fracture and nearby microcracks, and water had a softening effect on crack toughness, resulting in its expansion^36,37^. Under the combined effect of these factors, as water immersion intensified, the microstructure of coal samples underwent a progressive transformation, the number of internal alteration points within the coal increased, accompanied by a rise in etch pits, the mineral particles gradually tended to be rounded and smooth, the cementation between particles weakened, the expansion of cracks and fissures accelerated, and the strength of coal samples further deteriorated.
  Figure 7a reflects the variation between the total deterioration degree of compressive strength in coal samples and the number of water immersions. From Fig. 7a, it can be seen that the total deterioration degree of coal samples at 2 MPa, 4 MPa, 6 MPa, and 8 MPa was 32.80%, 29.73%, 26.31%, and 25.17%, respectively, after four times of water immersion. It indicated a clear decline in the compressive strength of coal samples when subjected to water immersion, and the total deterioration degree of the compressive strength of coal samples under different confining pressure increased with water immersion times, and the extent of deterioration was more pronounced at lower confining pressure. Furthermore, the total deterioration degree of the compressive strength of coal samples showed a certain level of non-uniformity as water immersion times increased. The total deterioration degree of compressive strength was the highest at 1 time of water immersion, and coal samples subjected to a confining pressure of 2 MPa experienced a total deterioration degree of compressive strength of 17.42%, after which the total deterioration degree gradually stabilized.
  Figure 7b reflects the relationship between the stage deterioration degree of the compressive strength of coal samples and the number of water immersions. The compressive strength deterioration law of coal samples during repeated water immersion has evident non-uniformity, as depicted in Fig. 7b. The stage deterioration degree was obviously larger when the coal sample was immersed once, and the stage deterioration degree of coal samples with a 2 MPa confining pressure reached 17.42%. The stage deterioration degree decreased significantly at the second immersion, and the stage deterioration degree of coal samples with 4 MPa confining pressure was reduced to 4.52%. After that, the stage deterioration degree exhibited a pattern of initial increase followed by decrease. The rate of deterioration trend of compressive strength decelerated and the stage deterioration degree reduced when the coal samples were immersed for 3–4 times, in which the stage deterioration degree of coal samples with 6 MPa confining pressure decreased to 2.04%. Furthermore, variation in the deterioration pattern of compressive strength can be observed among coal samples subjected to different levels of confining pressure. Specifically, coal samples exposed to higher confining pressure exhibited a relatively gradual decline in compressive strength, while those subjected to lower confining pressure experienced a more pronounced decrease in strength.
  From the above, the compressive strength deterioration effect of coal samples with different immersion times had obvious non-uniformity, which indicated that water had distinct physical, chemical and mechanical effects on coal at different immersion stages. Coal was hydrophilic and the physisorption of coal to water relied on intermolecular forces, which occured rapidly. In contrast, chemisorption, which necessitated the input of activation energy, proceeded at a slower rate^38^. Upon first immersion in water, coal underwent complete internal saturation, leading to the softening of its particle skeleton and dissolution of minerals and cements, resulting in a significant reduction in coal strength. Subsequently, with the repetition of the immersion process, the physical and mechanical effects of water on coal weakened, while the chemical effects gradually intensified and assumed a dominant role. Consequently, the strength of the coal steadily diminished and eventually reached a plateau. Under the combined effect of these factors, the stage deterioration degree of compressive strength of coal samples fell back significantly when soaked twice, and then showed a trend of initial increase followed by subsequent decrease. This indicated that the impact of water on the deterioration of coal exhibited a clear cumulative influence, resulting in different extents of deterioration at different stages of water immersion.
  Figure 8 reflects the relationship between the confining pressure and compressive strength of coal samples with different numbers of water immersions. From Fig. 8, it can be seen that the compressive strength of the soaked coal samples increased linearly with the increase in the confining pressure. For example, the strength of the dry coal sample (52.24 MPa) and the strength of the coal sample soaked once (43.96 MPa) under the confining pressure of 4 MPa were 9.13% and 10.83% higher than the strength of the dry coal sample (47.47 MPa) and the strength of the coal sample soaked once (39.20 MPa) under the confining pressure of 2 MPa, respectively. Combined with Figs. 6 and 7, the comprehensive analysis showed that the confining pressure had an enhancing effect on the strength of coal samples and can reduce the amount of deterioration of the compressive strength of coal samples to some extent. From one perspective, the presence of numerous micro-defects in coal led to their compression and closure under confining pressure. This compression restricted internal particle slippage and fracture and diminished the generation of shear deformation. Conversely, the water within coal generally resided within the crystalline layers of clay minerals and between the particles of these minerals. As confining pressure intensified, the thickness of the water film between particles decreased, causing water to flow elsewhere. Meanwhile, perpendicular to the confining pressure force between the crystal layer of water would also be pressed out into the original, not affected by the water of the clay particles or particles within the crystal layer, so that the local compressive strength decreased but the overall trend remained upward.
2. Analysis of the deterioration law of the cohesive force and internal friction angle of repeatedly immersed coal.
  From Fig. 8, it can be seen that the compressive strength is roughly linearly related to the confining pressure and satisfies the Mohr–Coulomb strength criterion, with the specific expression:
  4 $$\sigma_{1} { = }Q + K\sigma_{3}$$
  where: σ_1_ is the compressive strength, MPa; σ_3_ is the confining pressure, MPa; Q, K are the parameters of the material. Based on Eq. (4), the expressions for the relationship between internal friction angle and cohesive force with Q, K can be obtained as:
  5 $$\varphi = \arctan \frac{K - 1}{{2\sqrt K }}$$
  6 $$c = \frac{Q}{2\sqrt K }$$
  The triaxial compressive strength test data were fitted and analyzed using the Mohr–Coulomb criterion, and the internal friction angle and cohesive force of coal samples under different numbers of immersions can be calculated by combining Eqs. (5) and (6). The results were shown in Table 4. Figures 9 and 10 was made to visually analyze the properties of cohesive force, internal friction angle, and the deterioration law of coal samples under repeated water immersion.

**Table 4:** Internal friction angle and cohesive force of coal samples with different water immersion times.

Number of immersion (time)	Internal friction angle (°)	Cohesive force (MPa)
0	29.13	12.09
1	29.62	9.53
2	28.79	9.02
3	28.67	8.06
4	28.26	7.76

**Figure 9:**
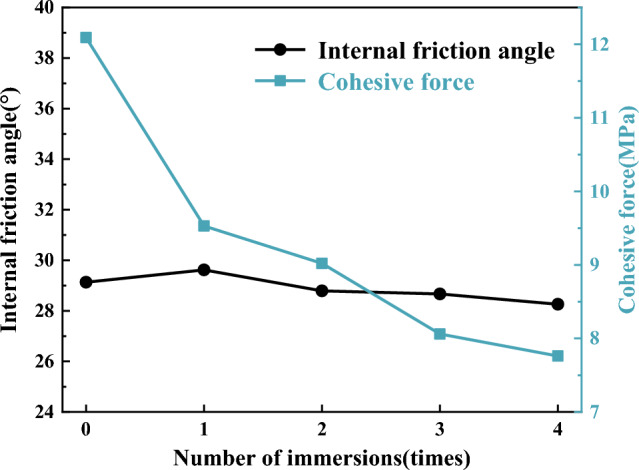
Graph of the internal friction angle and cohesive force of coal samples under different immersion times.

**Figure 10:**
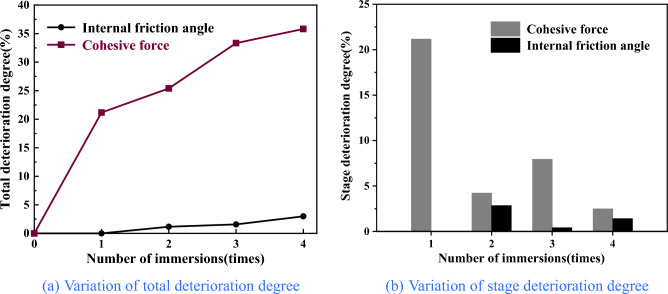
Diagram of deterioration degree of the internal friction angle and cohesive force of coal samples.
  Figure 9 reflects the variation of the internal friction angle and cohesive force of coal samples with the number of water immersions. The data presented in Fig. 9 demonstrated a drop in the cohesive force of coal samples from 12.09 to 7.76 MPa as the number of immersions increased from 0 to 4, resulting in a reduction of 35.81%. Additionally, the internal friction angle of the coal samples decreased from 29.13° to 28.26°, which decreased by 2.99%. It can be seen that the internal friction angle and cohesive force of coal samples decreased to different degrees with the increase in water immersion times, and the decrease in cohesive force was significantly larger than the change in internal friction angle.
  Figure 10a reflects the variation of the total deterioration degree of the internal friction angle and cohesive force of coal samples under different number of water immersions. From Fig. 10a, it was evident that the total deterioration of the internal friction angle and cohesive force of coal samples after 4 times of water immersion was 2.99% and 35.81%, respectively. It can be seen that the cohesive force of the coal sample significantly weakened when exposed to water immersion, while the internal friction angle experienced minimal deterioration and had negligible impact. The total deterioration of the cohesive force of the coal samples was the largest at the first water immersion, with the total deterioration degree reaching 21.17%, and then the increase in total deterioration degree gradually decreased with increasing water immersion times.
  Figure 10b reflects the variation of the stage deterioration degree of the internal friction angle and cohesive force of coal samples under different water immersion times. As depicted in Fig. 10b, similar to the deterioration law of compressive strength of coal samples, the deterioration law of internal friction angle and cohesive force of coal samples during repeated water immersion has evident non-uniformity. The stage deterioration degree of the cohesive force was significantly larger, reaching 21.17%, following a single immersion of the coal. The stage deterioration degree of cohesive force decreased significantly to 4.22% during the second immersion. After that, the stage deterioration degree of cohesive force showed a pattern of initial increase followed by decrease, and the deterioration trend of compressive strength slowed down. The stage deterioration degree became smaller when the coal sample was immersed for 3–4 times, and the stage deterioration degree of cohesive force decreased to 2.48% after the fourth immersion. The internal friction angle exhibited a pattern of initial increase followed by a decrease following the first immersion, whereas the stage deterioration reached its peak value of 2.85% after two immersions. It can be seen that the deterioration of the internal friction angle of the coal sample was relatively gentle, while the deterioration of the cohesive force was relatively large.
  The reason for this phenomenon was that the coal was relatively dense internally when it was first soaked in water, and the shear surface was formed mainly by irregular mineral particles staggering and shearing. As the number of immersions increased, the mineral particles tended to be rounded and smooth due to the water's influence, and the shear surface was formed mainly by rolling and sliding. Simultaneously, the dissolution of hydrophilic minerals and cement within the coal led to the augmentation of internal secondary porosity. This caused a gradual reduction in the area of internal interparticle cementation and strength, ultimately resulting in a loosening of the coal structure, and the cohesive force and internal friction angle of the coal sample gradually deteriorated. Among them, the cohesive force was mainly affected by the degree of cementation between mineral particles, which resulted in a faster deterioration rate. On the other hand, the internal friction angle was primarily influenced by the degree of mineral particle embedment and the strength of the particles themselves, leading to a relatively slower deterioration rate. In essence, the cohesive force of the coal sample was a structural parameter, while the angle of internal friction was a material parameter.

### Acoustic emission damage characteristics of repeatedly immersed coal

**Figure 11:**
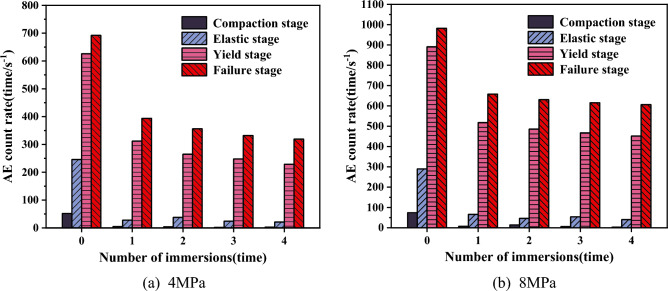
Histogram of average count rate of acoustic emission at each loading stage of coal samples.

**Figure 12:**
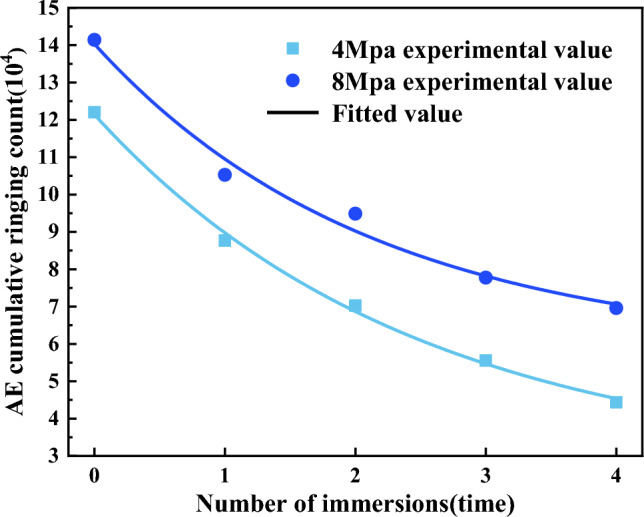
Acoustic emission cumulative ringing counts of coal samples with different water immersion times.

When the coal or rock is deformed by force, its internal primary fractures and defects expand, and the newborn micro-rupture keeps breeding, sprouting, evolving, expanding, and even fracturing, and the energy stored inside the coal or rock propagates outward in the form of elastic waves, forming acoustic emission signals. Acoustic emission count is a commonly used acoustic emission characterization parameter that reflects the energy released from the coal or rock due to the process of internal crack formation and expansion. The level of acoustic emission count indicates the magnitude of the internal damage to the coal or rock under external loading. Since the accumulated acoustic emission ringing counts and energy characteristics were similar in regularity, this paper only listed the results of the AE average count rate at each loading stage and the AE cumulative ringing count with different number of immersions under the confining pressure of 4 MPa and 8 MPa, as shown in Tables 5 and 6. To more intuitively reflect its relationship with the water immersion times, Figs. 11 and 12 was made.

**Table 5:** Statistics on the average count rate of acoustic emission at each loading stage of coal samples with different water immersion times.

Stage	Average AE count rate of coal samples (4 MPa)/(times s^−1^)					Average AE count rate of coal samples (8 MPa)/(times s^−1^)				
	Dry	Immersion				Dry	Immersion			
		1	2	3	4		1	2	3	4
Compaction stage	52	5	4	2	3	74	7	14	6	3
Elastic stage	246	28	38	24	21	290	66	46	54	40
Yield stage	626	312	265	248	229	891	518	486	467	452
Failure stage	692	394	356	332	319	982	658	631	615	606

**Table 6:** Acoustic emission cumulative ringing counts of coal samples with differe water immersion times.

Water immersion times	Acoustic emission cumulative ringing count (10^4^)	
	4 MPa	8 MPa
0	12.20	14.14
1	8.77	10.53
2	7.02	9.48
3	5.56	7.77
4	4.44	6.96

From Table 5 and Fig. 11, acoustic emission signals were generated during the compression damage process of coal samples, and the maximum duration of the AE count rate appeared in a very short time near the stress peak, but notable distinctions were observed between the test outcomes of dry state coal samples and water-soaked coal samples. During the compaction stage, the dry coal samples exhibited a limited quantity of acoustic emission signals with little variation, but the water-soaked samples had a near absence of acoustic emission signals. The average AE count rate of the dry coal samples was 2–26 times that of the water-soaked coal samples, mainly because the water-soaked coal samples were softened significantly by water and had a tendency to creep, so that the deformation and destruction of the softened coal samples were relatively weaker than that of the dry coal samples. During the elastic stage, the AE count rate of the dry-state coal samples and the water-soaked coal samples began to increase, and it was relatively steady. Entering the yielding stage, the acoustic emission signals of both the dry coal samples and the water-soaked coal samples increased significantly. Comparison with the elastic stage, the average AE count rate in the coal samples during the yielding stage was found to be 3–11 times higher. Entering the failure stage, the fissure penetration near the peak load produced the main rupture and reached the maximum value of the count rate. Due to the presence of water to reduce the strength of coal, compared with the dry state of the coal samples, the average AE count rate of coal samples immersed in water for 1 time decreased significantly. However, subsequent immersions (2, 3, and 4 times) resulted in only marginal decrease in the average AE count rate. This suggested that the influence of the number of immersions on the acoustic emission characteristics tended to stabilize after a certain threshold.

Figure 12 shows the AE cumulative ringing counts of coal samples under different water immersion times. From Table 6 and Fig. 12, the AE cumulative ringing counts of coal samples exhibited the highest values at 0 times of immersion, measuring 12.20 × 10^4^ and 14.14 × 10^4^, respectively. Conversely, the AE cumulative ringing counts of coal samples were the smallest at 4 times of immersion, measuring 4.44 × 10^4^ and 6.96 × 10^4^, respectively, with a decrease of 63.61% and 50.78% compared with those before immersion. The coal samples exhibited the most significant reduction in the AE cumulative ringing count during the first water immersion, with a decrease of 28.11% and 25.53%, and then the rate of decline gradually diminished until it eventually stabilized. The phenomenon can be attributed to the infiltration of water into the internal fissures of coal, weakening the interparticle linkage and reducing the strength of coal particles and particle adhesion. Consequently, the energy required for coal rupture was reduced, leading to a weakening of the acoustic emission activity during the compression process and a subsequent decrease in the AE cumulative ringing counts. Table 7 depicted the fitted relationship between the AE cumulative ringing count of coal samples and the water immersion times.

**Table 7:** Fitted relationship between the acoustic emission cumulative ringing count of coal samples and water immersion times.

Confining pressure	Fitting equation	R^2^
4 MPa	y = 9.457*exp(− x/2.454) + 2.682	0.9976
8 MPa	y = 8.224*exp(− x/2.131) + 5.805	0.9868

## Discussion

Coal, being a naturally occurring composite material that possesses small pores and fissures, exhibits a high degree of sensitivity to its inherent original flaws. Both repeated water immersion and mechanical loading cause the deterioration of the strength, elastic modulus, and other mechanical properties of coal. However, the analysis of previous test results shows that the two damage mechanisms are distinct. Repeated water immersion changes the fine structure of the coal body, leading to an increase in the distribution of pore cleavage and a decrease in the density of impurities. On the other hand, mechanical loading is known to cause macroscopic damage to the coal body, while the coal particles themselves remain undamaged, even the very fine coal particles. Repeated water immersion plays a deteriorating role on the coal body, while the coal body may experience a strengthening effect when subjected to loading, resulting in an increase in both its strength and elastic modulus under the influence of confining pressure. This study employs the principles of damage mechanics theory to incorporate damage variables that effectively describe the extent and condition of coal with microscopic imperfections, and a comprehensive damage model for coal bodies is established. Given *D* representing the total damage of a coal body subjected to both repeated water immersion and mechanical loading, the damage variable under the only condition of mechanical loading can be denoted as *D*_*σ*_, while the damage variable under repeated water immersion alone can be denoted as *D*_*n*_.

7 $$D = \frac{{A_{t} }}{A}$$

8 $$D_{\sigma } = \frac{{A_{1} }}{A}$$

9 $$D_{n} = \frac{{A_{2} }}{A}$$

where: *A* is the initial undamaged cross-sectional area, *A*_*t*_ is the total cross-sectional area of the damaged part of the coal body, *A*_1_ is the cross-sectional area of the damaged part by stress, *A*_2_ is the cross-sectional area of the damaged part by water. Separate consideration of the stress and water action can be given to the fundamental unit body of the coal body during analysis. Considering firstly the action of water damage, its damaged density is $D_{n} = \frac{{A_{2} }}{A}$; after considering the average damaged area *A*_1_ under the action of stress damage, its damaged density is $D_{\sigma } = \frac{{A_{1} }}{A}$, then the cross-sectional area *A*_*d*_ of the total damaged portion of the coal body is:

10 $$A_{t} = \frac{{A_{2} }}{A} \cdot A + \frac{{A_{1} }}{A}\left( {A - A_{2} } \right) = A_{1} + A_{2} - \frac{{A_{1} A_{2} }}{A}$$

Therefore, the repeated immersion-stress coupling damage variable can be defined as:

11 $$D = \frac{{A_{t} }}{A} = \frac{{A_{1} }}{A} + \frac{{A_{2} }}{A} - \frac{{A_{1} A_{2} }}{{A^{2} }} = D_{\sigma } + D_{n} - D_{\sigma } D_{n}$$

**Figure 13:**
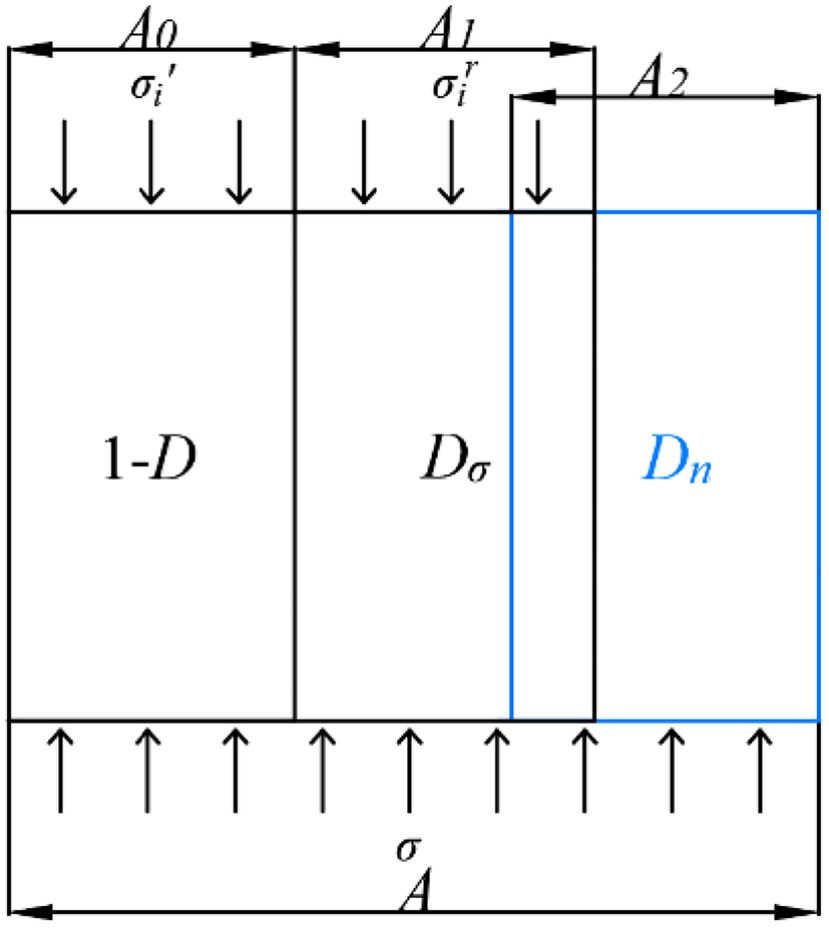
Coal body damage model considering repeated water immersion effects.

Based on this, this paper proposes a coal body damage model considering repeated water immersion, as shown in Fig. 13, which needs to satisfy the following conditions:

1. The internal porosity of the coal body has a random distribution, whereas its mechanical characteristics demonstrate isotropy.
2. Coal body damage is limited to the axial direction, with no damage occurring in the lateral direction. This means that the load experienced by the coal body in the axial direction is distributed between the damaged and undamaged parts, and the nominal stress and effective stress of the coal body in the lateral direction are equivalent.
3. Damage to the coal body means destruction, and the axial stress applied rapidly reaches the residual strength, i.e., the axial stress applied to the damaged part of the coal body is the residual stress strength $\sigma_{i}^{r}$.
4. The intact portion of the material is composed of an elastomer that adheres to the principles outlined in the generalized Hooke's law, specifically:
  12 $$\sigma_{i}^{\prime } = E\varepsilon_{i}^{\prime } + \mu \left( {\sigma_{j}^{\prime } + \sigma_{k}^{\prime } } \right)$$
  where: $\sigma_{i}^{\prime }$ (i, j, k = 1, 2, 3) is the effective stress on the undamaged part of the coal body;* E* and $\mu$ are the elastic modulus and Poisson's ratio of the intact coal body, respectively; $\varepsilon_{i}^{\prime }$ is the strain of the undamaged part of the coal body.

From the strain-equivalence hypothesis proposed by Lemaitre^39^, the deformation behavior of damage-containing materials can be realized by effective stress, that is, the strain relation of damaged materials can be expressed in the form when they are undamaged, by simply replacing the nominal stress with the effective stress, then:

13 $$\sigma_{i} = \sigma_{i}^{\prime } \left( {1 - D} \right)$$

where: $\sigma_{i}$ is the nominal stress on the damaged material, $\sigma_{i}^{\prime }$ is the effective stress on the undamaged material, and *D* is the damage variable.

This hypothesis assumes that microfractures are formed within the material when damage occurs and that these microfractures are unable to withstand any stress. The resulting coal damage model makes it difficult to characterize the residual strength of the coal after damage. When the coal reaches complete damage, its damage variable *D* = 1, and the nominal stress on the coal = 0, which is obviously inconsistent with the reality, and the coal still has a certain residual bearing capacity after complete damage.

Based on this, Cao et al.^40^ proposed an improved novel damage model for coal and rock:

14 $$\sigma_{i} = \sigma_{i}^{\prime } \left( {1 - D} \right) + \sigma_{i}^{r} D$$

where: $\sigma_{i}^{r}$ is the axial residual strength of the coal and rock, the magnitude of which does not change with axial deformation.

According to the above assumptions and combining Eq. (12), Eq. (14), the damage constitutive model of the coal body under triaxial compression condition can be obtained as:

15 $$\sigma_{1} = \left[ {E\varepsilon_{1} + \mu \left( {\sigma_{2} + \sigma_{3} } \right)} \right]\left( {1 - D} \right) + \sigma_{1}^{r} D$$

Substituting Eq. (11) into Eq. (15), then:

16 $$\sigma_{1} = \left[ {E\varepsilon_{1} + \mu \left( {\sigma_{2} + \sigma_{3} } \right)} \right]\left( {1 - D_{\sigma } } \right)\left( {1 - D_{n} } \right) + \sigma_{1}^{r} \left( {D_{\sigma } + D_{n} - D_{\sigma } D_{n} } \right)$$

The multiplication result of the residual effective stress $\sigma_{1}^{r}$ and the damage variable *D*_*n*_ under repeated immersion is 0. Under the general triaxial condition ($\sigma_{2} = \sigma_{3}$), the coal damage constitutive model considering repeated immersion is as follows:

17 $$\sigma_{1} = \left[ {E\varepsilon_{1} + 2\mu \sigma_{3} } \right]\left( {1 - D_{\sigma } } \right)\left( {1 - D_{n} } \right) + \sigma_{1}^{r} D_{\sigma }$$

Since the microfracture evolution of coal samples is a random variation, the fracture evolution of coal samples can be viewed as a non-equilibrium statistical process, and the micro-unit intensity distribution is considered to obey the Weibull distribution, and its distribution density function is as follows^41^:

18 $$P(F) = \frac{m}{{F_{0} }}\left( {\frac{F}{{F_{0} }}} \right)^{m - 1} \exp \left[ { - \left( {\frac{F}{{F_{0} }}} \right)^{m} } \right]$$

where: *F* is the random distribution variable of the Weibull distribution of the micro-unit strength of the coal sample; m and *F*_*0*_ are the Weibull distribution parameters characterizing the physical and mechanical properties of the coal sample.

Coal samples subjected to external loading produce energy accumulation, and the accumulated strain energy is released outward in the form of elastic waves, producing an acoustic emission phenomenon. In essence, the rule of acoustic emission activity is a statistical rule, which necessarily coincides with the rule of statistical distribution of damage inside the coal sample. Heiple et al. concluded that the acoustic emission ringing count is one of several parameters characterizing the acoustic emission signal that better reflects the damage properties of the material, which is proportional to the movement of dislocations in the material, the exfoliation and fracture of inclusions and second-phase particles, and the strain energy released by crack extension^42^.

Kachanov defines the damage variable as:

19 $$D = \frac{{A_{d} }}{A}$$

where: *A*_*d*_ is the area of all micro-defects in the bearing section, and *A* is the area of the section when it is initially undamaged.

If the acoustic emission cumulative ringing count when the entire section A of the undamaged coal sample is completely destroyed is *N*_*f*_, then the acoustic emission cumulative ringing count *N*_0_ for destruction of micro-units per unit area of the coal sample during compression is as follows:

20 $$N_{0} = \frac{{N_{f} }}{A}$$

When the sectional damage area reaches *A*_*d*_, the acoustic emission cumulative ringing count is:

21 $$N_{d} = N_{0} \cdot A_{d} = N_{f} \cdot \frac{{A_{d} }}{A}$$

Combining Eqs. (19) and (21), the relationship between damage variable and acoustic emission cumulative counts can be derived as:

22 $$D = \frac{{N_{d} }}{{N_{f} }}$$

From Eq. (22), it can be seen that the acoustic emission can adequately characterize the damage degree of coal samples during the compression process. The AE cumulative count of coal samples decreases with the increase in the water immersion times, and it can be seen that the AE cumulative count and the number of immersions conform to the relational equation *y* = A*exp(− x/*B*) + *C* by fitting the equation. Setting the initial damage variable of the coal samples that are undamaged to be 0 and the damage variable of the entire cross section A to be 1 once it is completely damaged, then:

23 $$D_{\sigma } = 1 - \frac{{A\exp \left( { - \frac{n}{B}} \right) + C}}{{N_{f} }}$$

Coal samples subjected to repeated water immersion produce microscopic fractures, the fracture networks develop, and the elastic modulus gradually decreases with the increase of water immersion times. The "elastic modulus method" in the continuous medium damage theory is a method based on the strain-equivalence hypothesis, which defines or measures the damage by the variation of the elastic modulus before and after the material damage^43^. According to the damage definition, the elastic modulus can be used to the define damage variable *D*_*n*_ when considering the water immersion damage alone:

24 $$D_{n} = 1 - \frac{{E_{n} }}{{E_{0} }}$$

where: $E_{0}$ is the elastic modulus of the dry coal sample, $E_{n}$ is the elastic modulus of the coal sample when the water immersion times is n.

**Figure 14:**
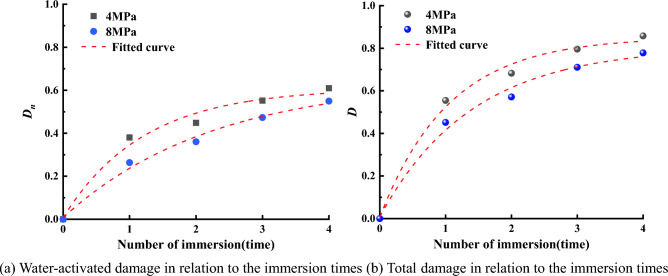
Evolution curve of coal sample damage with the water immersion times.

Combining Eqs. (11), (23) and (24), Fig. 14 was made. Through fitting, it was found that *D*_*n*_, *D* and the number of immersions were consistent with the relationship of *y* = A*exp(− x/*B*) + *C*, as shown in Tables 8 and 9. The data presented in Fig. 14 clearly indicated that as the water immersion times increased, the extent of water-induced damage to coal also increased, but the increase gradually decreased. The rate of coal damage caused by immersion exhibited a gradual decrease as the number of immersions increased. Notably, the initial immersion of coal in water resulted in more significant damage, characterized by a higher damage rate. However, as the number of immersions increased, the detrimental effects of water on coal became less pronounced. This suggested that the impact of water damage on coal reached a state of stability once a certain threshold of immersion times was reached.

**Table 8:** Water action damage variable *D*_*n*_ of coal samples as a function of the number of immersions.

Confining pressure	D_n_ as a function of the number of immersions	R^2^
4 MPa	y = − 0.601*exp(− x/1.209) + 0.608	0.9831
8 MPa	y = − 0.639*exp(− x/2.248) + 0.647	0.9920

**Table 9:** Total damage variable *D* of coal samples as a function of the number of immersions.

Confining pressure	D as a function of the number of immersions	R^2^
4 MPa	y = − 0.846*exp(− x/1.054) + 0.852	0.9927
8 MPa	y = − 0.798*exp(− x/1.399) + 0.806	0.9905

In view of the above study, water storage in underground reservoirs of coal mines will produce erosion damage to coal pillar dams and affect the structural strength of the dams, among which the deterioration of the mechanical properties of the coal pillar dams during the initial water immersion is the greatest. Therefore, it is necessary to drain the coal pillar as early as possible during the coal mining process to reduce the water damage to the coal pillar, and at the same time, the stress and displacement of the coal pillar should be monitored in the underground reservoir storage to ensure the stability of the coal pillar dam after the initial immersion.

## Conclusion

1. The saturated water content of coal samples increased continuously with the increase in water immersion times, albeit at a diminishing rate of growth. The deterioration of the compressive strength and cohesive force of coal samples under repeated immersion was obvious, whereas the reduction in internal friction angle was rather minor. These effects had obvious cumulativity and non-uniformity. With the increase in water immersion times, the compressive strength, cohesive force, and internal friction angle of coal samples decreased to different degrees, and the total deterioration degree increased continuously but gradually decreased. The stage deterioration degree fell back significantly at two times of water immersion and then showed a trend of initial increase followed by decrease. The coal sample exhibited the highest level of deterioration during the initial immersion, followed by a progressive decline and subsequent stabilization.
2. The variation in the average AE count rate and the AE cumulative ringing counts of coal samples loaded with different numbers of immersions were analyzed. The average AE count rate at each stage of loading and the AE cumulative ringing count of coal samples dropped as the water immersion times increased. The internal microstructure of coal underwent changes due to the water–rock interaction, resulting in a significant decrease in the strength of coal particles and particle adhesion, and the acoustic emission activity was weakened.
3. Based on the damage theory, a coal damage model considering the water immersion times was established, and the evolution curve of coal damage with the water immersion times under the hydrodynamic coupling was obtained. As the increase of the number of immersions, the damage of water on coal also increased gradually, but with a diminishing rate of increase. The damage and deterioration of coal samples were especially significant during the first water immersion.
4. The mechanisms of coal deterioration under water–rock interaction were discussed. During repeated water immersion, the physical, chemical, and mechanical effects of water on coal led to internal microstructural changes in coal samples, resulting in the deterioration of mechanical properties such as compressive strength, cohesive force, and internal friction angle, which was a cumulative damage process from microscopic to macroscopic.

## Data Availability

All data generated or analyzed during this study are included in this published article.
